# Application of Overlap Gastroduodenostomy in Billroth I Anastomosis after Totally Laparoscopic Distal Gastrectomy for Gastric Cancer

**DOI:** 10.1155/2022/9094934

**Published:** 2022-08-12

**Authors:** Guojun Chen, Wenhuan Li, Weihua Yu, Dong Cen, Xianfa Wang, Peng Luo, Jiafei Yan, Guofu Chen, Yiping Zhu, Linhua Zhu

**Affiliations:** ^1^Department of Gastrointestinal Surgery, Sir Run Run Shaw Hospital, Zhejiang University School of Medicine, Hangzhou 310016, China; ^2^Department of Gastrointestinal Surgery, The First People's Hospital of Wenling, Taizhou 317500, China

## Abstract

Delta-shaped gastroduodenostomy (DSGD) and overlap gastroduodenostomy (OGD) are the two most widely used intracorporeal Billroth I anastomosis methods after distal gastrectomy. In this study, we compared the short-term outcomes of DSGD and OGD in total laparoscopic distal gastrectomy (TLDG). In a retrospective cohort study, we examined 92 gastric cancer patients who underwent TLDG performed by the same surgeon between January 2014 and June 2018. All patients underwent Billroth I reconstruction (OGD, *n* = 45; DSGD, *n* = 47) and D2 lymph node dissection. We retrospectively reviewed the surgical outcomes, clinical pathological results, and endoscopy results. Laparoscopic surgery was successfully performed in both groups without conversion to open surgery. The demographic and clinical characteristics were similar between the two groups (*P* > 0.05). There were no significant differences between the two groups in operation time (158.9 ± 13.6 min vs. 158.8 ± 14.8 min, *P*=0.955), anastomotic time (19.4 ± 3.0 min vs. 18.8 ± 2.9 min, *P*=0.354), intraoperative blood loss (88.9 ± 25.4 mL vs. 83.7 ± 24.3 mL, *P*=0.321), number of lymph node dissections (31.0 ± 7.1 vs. 29.2 ± 7.5, *P*=0.229), length of hospital stay (8.8 ± 2.7 days vs. 9.1 ± 3.0 days, *P*=0.636), fluid intake time (3.1 ± 0.7 days vs. 3.2 ± 0.7 days, *P*=0.914), and morbidity of postoperative complications (6.7% [3/45] vs. 10.6% [5/47], *P*=0.499). Endoscopy performed 6 months postoperatively showed that the residual food (*P*=0.033), gastritis (*P*=0.029), and bile (*P*=0.022) classification score significantly decreased in the OGD group, and there were no significant differences 12 months postoperatively. OGD is a safe and effective reconstruction technique with comparable postoperative surgical outcomes and endoscopy results when compared with those of DSGD.

## 1. Introduction

Gastric cancer (GC) is one of the most common malignancies in China [1]. The incidence of the early detection of GC has increased significantly with an increase in the frequency of endoscopic screening. Surgical resection remains the only curative treatment. Laparoscopic gastrectomy has been widely used since it was first reported in 1994 by Kitano et al. [2]. In recent years, an increasing number of clinical studies have shown that laparoscopic gastrectomy has similar or better outcomes than open gastrectomy [3–6].

Owing to the narrow and restricted space, it is difficult to perform anastomosis in laparoscopic-assisted distal gastrectomy (LADG), especially in obese patients or in patients with a small remnant stomach. Therefore, many surgeons prefer total laparoscopic distal gastrectomy (TLDG), and several techniques for intracorporeal anastomosis have been developed [7].

Billroth I, Billroth II, and Roux-en-Y are the three most commonly used reconstruction methods after distal gastrectomy, and Billroth I gastroduodenostomy is the only method that retains the physiological digestive tract and poses no risk of internal hernia. Delta-shaped gastroduodenostomy (DSGD), which was first introduced by Kanaya et al. [8], is the most popular anastomosis method in Billroth I reconstruction after TLDG overlap gastroduodenostomy (OGD), first introduced by Song et al. [9] and modified by Byun et al. [10], appears to be another simple and convenient method for Billroth I reconstruction after TLDG. In this study, we assess the short-term results of OGD and compare them with those of DSGD.

## 2. Materials and Methods

### 2.1. Materials

This single-surgeon retrospective cohort study was performed between January 2014 and June 2018 at six different hospitals in Zhejiang Province, China. The inclusion criteria were as follows: (1) preoperative diagnosis of cT1N0M0∼cT2N0M0-stage GC and (2) postoperative pathology confirming R0 resection. All perioperative management procedures were performed under the surgeon's guidance.

According to the above criteria, 92 patients were included in this retrospective study, including 47 with DSGD and 45 with OGD. As the surgeon changed the main reconstruction method when performing Billroth I anastomosis, DSGD was mainly completed between January 2014 and September 2016, and OGD was mainly completed between September 2016 and June 2018.

### 2.2. Surgical Procedure

Under general anesthesia, the patients were placed in the reverse Trendelenburg position at approximately 30°. Pneumoperitoneum was established with CO_2_ at a pressure of 11–13 mmHg. We placed five ports in V-shape, and at the vertex position was a 10-mm camera port. The other four working ports were placed in the right upper quadrant (5 mm), right middle quadrant (5 mm), left middle quadrant (5 mm), and left upper quadrant (12 mm) of the abdomen.

Lymph node dissection and omentectomy were performed according to Japanese guidelines. The resection lines of the duodenum and stomach were determined according to the tumor site. We used a 60-mm endoscopic linear stapler to perform resection and reconstruction in the OGD group and a 45-mm endoscopic linear stapler in the DSGD group.

### 2.3. DSGD

We modified the DSGD to be similar to that reported by Huang et al. [11] We rotated the duodenum 90° clockwise when it was transected, and a small incision was made on the posterior side of the greater curvature of the remnant stomach and on the posterior side of the duodenum. Then, we inserted the linear stapler, closed and fired it, and created a V-shaped anastomosis on the posterior wall. After verifying the absence of bleeding from the anastomosis, we closed the common stab incision along the blind angle of the duodenum.

### 2.4. OGD

There is no need to perform a 90° rotation when the duodenum is transected. A small incision was made on the greater curvature of the remnant stomach, and another small incision was created on the superior edge of the duodenal transection line. The linear stapler was introduced into the remnant stomach and duodenum, where the two sides were put together, and the stapler was closed and fired. After verifying that there was no bleeding on the stapler line, a one-stay suture was added in the middle of the common stab incision. We then pulled the stay suture and two ends of the common stab incision and closed the incision using the linear stapler.

### 2.5. Postoperative Follow-Up Evaluation

All patients were managed in a similar manner, following the postoperative clinical path. The gastric tube was removed on postoperative day 1, and water intake was initiated on postoperative day 2.

Monitoring indicators included operation time, anastomotic time, intraoperative blood loss, number of lymph node dissections, length of hospital stay, fluid intake time, and complications. Regular follow-ups were conducted 3, 6, 9, and 12 months postoperatively, and endoscopic examinations were performed in the first 6 and 12 months postoperatively. Endoscopic findings were evaluated using the residual food, gastritis, and bile (RGB) classification [12].

### 2.6. Statistical Analysis

All statistical analyses were performed using the Statistical Package for the Social Sciences (SPSS), version 20.0 for Windows (SPSS Inc., Chicago, United States). Data are expressed as mean ± standard deviation. Categorical variables were analyzed using the chi-square test or Fisher's exact test, while continuous variables were analyzed using Student's *t*-test. Statistical significance was set at *P* < 0.05.

## 3. Results

### 3.1. Clinicopathological Characteristics

The clinicopathological characteristics of the two groups are summarized in Table 1; 45 patients received OGD, and 47 patients received DSGD. No significant differences in age, sex, body mass index, history of abdominal surgery, tumor size, and TNM stage were found between the two groups.

### 3.2. Operative and Postoperative Characteristics

Laparoscopic surgery was successfully completed in all patients without conversion to open surgery. As Table 2 shows, there were no significant differences between the two groups in operation time (158.9 ± 13.6 min vs. 158.8 ± 14.8 min, *P*=0.955), anastomotic time (19.4 ± 3.0 min vs. 18.8 ± 2.9 min, *P*=0.354), intraoperative blood loss (88.9 ± 25.4 mL vs. 83.7 ± 24.3 mL, *P*=0.321), number of lymph node dissections (31.0 ± 7.1 vs. 29.2 ± 7.5, *P*=0.229), length of hospital stay (8.8 ± 2.7 days vs. 9.1 ± 3.0 days, *P*=0.636), fluid intake time (3.1 ± 0.7 days vs. 3.2 ± 0.7 days, *P*=0.914), and morbidity of postoperative complication (6.7% [3/45] vs. 10.6% [5/47], *P*=0.499).

The types of complications were comparable between the two groups. Two patients (4.4%) in the OGD group and one patient (2.1%) in the DSGD group developed delayed gastric emptying. All three of them were placed under conservative management. In the OGD group, the two patients were discharged on postoperative days 25 and 30, while, in the DSGD group, the patient was discharged on postoperative day 33. Two patients (4.3%) in the DSGD group had anastomotic leakage, whereas no patient had leakage in the OGD group. No in-hospital mortality was observed in either group.

### 3.3. Endoscopic Findings

Table 3 shows the endoscopic findings of the two groups 6 and 12 months postoperatively. Six months postoperatively, the RGB classification scores in the OGD group were significantly lower than those in the DSGD group (*P*=0.033, *P*=0.029, *P*=0.022, respectively). Twelve months postoperatively, there were no significant RGB classification score differences.

## 4. Discussion

TLDG, in which all procedures including gastric resection and digestive tract reconstruction are performed intracorporeally without making an additional abdominal incision, has become much more acceptable to surgeons because of its advantages over LADG [13, 14].

Currently, DSGD is the most popular reconstruction approach for Billroth I following TLDG. Although it is reported that DSGD has a satisfactory result and a relatively short learning curve [15], the rate of anastomosis-related complications is still relatively high [16–20]. The most important reason for this is that in DSGD, the surgeon must rotate the duodenal stump and the remnant stomach. However, these actions are mandatory procedures, and insufficient rotation might leave ischemic tissue between the transection lines and the anastomosis line. The OGD method has no such problems because it is a side-to-side overlap anastomosis, and duodenal rotation is unnecessary, thus reducing the possibility of damage to the surrounding structures and anastomotic ischemia. In our study, we had two cases of leakage in the DSGD group, while there was no case of leakage in the OGD group, although the difference was not significant.

In the OGD procedure, attention should be paid to the complications of delayed gastric emptying. We had two cases (4.4%) that resulted in delayed gastric emptying, and although all of them were cured after conservative treatment, the hospital stays were prolonged and the costs increased. The reason for this is not well known, and it might be because more stomach is retained, especially at the greater curvature, to insert the linear stapler and perform the anastomosis. All patients underwent D2 lymph node dissection, which may have influenced the blood supply to the remnant stomach, especially with a larger remnant stomach. This lack of blood supply might have caused delayed gastric emptying [21].

Regarding endoscopic findings based on the RGB classification, OGD was significantly lower 6 months postoperatively, but these differences disappeared 12 months postoperatively. This is probably owing to two reasons. First, the anastomosis in DSGD was posterior with rotation of the duodenum, which could then be twisted when food passed. Meanwhile, the anastomosis in OGD is a morphological “up and down” reconstruction where gastric content can pass easily. Second, in OGD, we used a 60-mm linear stapler (compared with the 45-mm stapler used in DSGD) that resulted in a larger anastomosis lumen and therefore faster passing of gastric content into the duodenum. OGD may thereby reduce the incidence of gastritis and bile reflux. In the DSGD group, these endoscopic findings improved from 6 to 12 months, which Lee et al. [22] have previously found, whereas, in the OGD group, they were almost the same.

Our study has some limitations. First, it was a retrospective cohort study, and the surgeon changed the main reconstruction method from DSGD to OGD. Although the surgeon had 12 years of experience in gastrectomy and 8 years of experience in laparoscopic gastrectomy, the surgical skill of reconstruction and D2 lymphadenectomy may have improved, which might have caused a bias. Second, our results came from only one surgeon without representing others; thus, a multicenter prospective study is needed. Finally, our study lacks long-term data. To evaluate postoperative outcomes, administering questionnaires would strengthen our findings.

## 5. Conclusion

Overlap gastroduodenostomy is a safe, simple, and feasible approach to intracorporeal anastomosis and has the same short-term results as delta-shaped gastroduodenostomy. However, long-term comparative studies are required for further assessment.

## Figures and Tables

**Table 1:** Clinicopathological characteristics of patients.

Characteristics	OGD (*n* = 45)	LSGD (*n* = 47)	*P* value
Age (years, $\bar{x}\pm s$)	59.1 ± 9.3	60.79 ± 11.6	0.435

Gender (no. %)			0.666
Male	25 (55.6)	24 (51.1)	
Female	20 (44.4)	23 (48.9)	

BMI (kg/m^2^, $\bar{x}\pm s$)	22.9 ± 2.8	23.2 ± 2.5	0.543
Previous abdominal surgery (no. %)	5 (11.1)	7 (14.9)	0.590
Tumor size (cm, $\bar{x}\pm s$)	2.4 ± 0.8	2.6 ± 0.8	0.301

T classification (no. %)			0.333
T1	25 (55.6)	19 (40.4)	
T2	19 (42.2)	26 (55.3)	
T3	1 (2.2)	2 (4.3)	

N classification (no. %)			0.516
N0	17 (37.8)	11 (23.4)	
N1	19 (42.2)	25 (53.2)	
N2	8 (17.8)	10 (21.3)	
N3	1 (2.2)	1 (2.1)	

**Table 2:** Operative and postoperative characteristics.

Characteristics	OGD (*n* = 45)	LSGD (*n* = 47)	*P* value
Operation time (min, $\bar{x}\pm s$)	158.9 ± 13.6	158.8 ± 14.8	0.955
Anastomotic time (min, $\bar{x}\pm s$)	19.4 ± 3.0	18.8 ± 2.9	0.354
Blood loss (ml, $\bar{x}\pm s$)	88.9 ± 25.4	83.7 ± 24.3	0.321
Retrieved lymph nodes (n, $\bar{x}\pm s$)	31.0 ± 7.1	29.2 ± 7.5	0.229
Length of stay (days, $\bar{x}\pm s$)	8.8 ± 2.7	9.1 ± 3.0	0.636
Liquid diet buildup (days, $\bar{x}\pm s$)	3.1 ± 0.7	3.2 ± 0.7	0.914
Any complication (no. %)	3 (6.7)	5 (10.6)	0.499
Wound infection	0 (0.0)	1 (2.1)	0.323
Leakage	0 (0.0)	2 (4.3)	0.160
Delayed gastric emptying	2 (4.4)	1 (2.1)	0.537
Pulmonary	1 (2.2)	1 (2.1)	0.976

**Table 3:** Endoscopic findings in postoperative 6 and 12 months.

Characteristics	6 months			12 months		
	OGD (*n* = 45)	LSGD (*n* = 47)	*P* value	OGD (*n* = 45)	LSGD (*n* = 47)	*P* value
Residual food (no. %)			0.033			0.194
Grade 0	28 (62.2)	15 (31.9)		25 (55.6)	16 (34.0)	
Grade 1	5 (11.1)	12 (25.5)		6 (13.3)	11 (23.4)	
Grade 2	5 (11.1)	4 (8.5)		5 (11.1)	4 (8.5)	
Grade 3	4 (8.9)	8 (17.0)		6 (13.3)	8 (17.0)	
Grade 4	3 (6.7)	8 (17.0)		3 (6.7)	8 (17.0)	

Gastritis (no. %)			0.029			0.072
Grade 0	5 (11.1)	3 (6.4)		5 (11.1)	3 (6.4)	
Grade 1	30 (66.7)	20 (42.6)		28 (62.2)	22 (46.8)	
Grade 2	8 (17.8)	22 (46.8)		8 (17.8)	20 (42.6)	
Grade 3	2 (4.4)	2 (4.3)		4 (8.9)	2 (4.3)	

Bile reflux (no. %)			0.022			0.168
Grade 0	28 (62.2)	18 (38.3)		26 (57.8)	19 (40.4)	
Grade 1	17 (37.8)	29 (61.7)		12 (26.7)	17 (36.2)	

## Data Availability

The data used to support the findings of this study were supplied by Linhua Zhu under license and so cannot be made freely available. Requests for access to these data should be made to Linhua Zhu, the e-mail address is zhulh@srrsh.com.
